# GOnet: a tool for interactive Gene Ontology analysis

**DOI:** 10.1186/s12859-018-2533-3

**Published:** 2018-12-07

**Authors:** Mikhail Pomaznoy, Brendan Ha, Bjoern Peters

**Affiliations:** 1 Department of Vaccine Discovery, La Jolla Institute for Allergy and Immunology, La Jolla, CA USA; 2 Department of Medicine, University of California San Diego, La Jolla, CA 92093 USA

**Keywords:** Gene ontology, GSEA, Interactive, Web-app, Genomics, Proteomics, Data analysis

## Abstract

**Background:**

Biological interpretation of gene/protein lists resulting from -omics experiments can be a complex task. A common approach consists of reviewing Gene Ontology (GO) annotations for entries in such lists and searching for enrichment patterns. Unfortunately, there is a gap between machine-readable output of GO software and its human-interpretable form. This gap can be bridged by allowing users to simultaneously visualize and interact with term-term and gene-term relationships.

**Results:**

We created the open-source GOnet web-application (available at http://tools.dice-database.org/GOnet/), which takes a list of gene or protein entries from human or mouse data and performs GO term annotation analysis (mapping of provided entries to GO subsets) or GO term enrichment analysis (scanning for GO categories overrepresented in the input list). The application is capable of producing parsable data formats and importantly, interactive visualizations of the GO analysis results. The interactive results allow exploration of genes and GO terms as a graph that depicts the natural hierarchy of the terms and retains relationships between terms and genes/proteins. As a result, GOnet provides insight into the functional interconnection of the submitted entries.

**Conclusions:**

The application can be used for GO analysis of any biological data sources resulting in gene/protein lists. It can be helpful for experimentalists as well as computational biologists working on biological interpretation of -omics data resulting in such lists.

## Background

The output of genome-wide studies is typically a list of genes (or their protein products) exhibiting a shared pattern. For example, these can be genes that are differentially expressed in groups of donors with and without a disease or a list of proteins identified by mass-spectrometry in a certain fraction of a biological sample. Making scientific sense out of such data is a complicated task requiring biological knowledge of the involved genes/proteins and their functions. As published data expands it becomes increasingly difficult to stay up to date with the constantly expanding knowledge and computational methods. Database resources become an important facility to make this knowledge accessible. The Gene Ontology (GO, http://geneontology.org/, [1]) is one such pioneering project, which maintains a controlled hierarchical vocabulary of terms along with logical definitions to describe molecular functions, biological processes, and cellular components. This controlled vocabulary is utilized by several model organism databases to capture experimental (and computational) findings on the role specific genes play. This knowledge can be applied to a given list of genes (also referred to as a gene-set) to explore the GO terms annotating the genes and to split them into functional groups (**‘annotation’** analysis). This approach is implemented, for example, in DAVID tool [2]. Another common step is to focus only on terms significantly over-represented in a list of entries submitted by a user (**‘enrichment’** analysis). This approach is a particular case of GSEA (gene set enrichment analysis) applied to Gene Ontology annotations. Such analysis can be carried out from the GO project website [3], using other web applications (e.g. GOrilla [4], NaviGO [5], DAVID [2], AmiGO [6]) or if a programmatic approach is needed one can use available modules for Python (e.g. GOATools [7], goenrich [8]) and R (e.g. GOstats [9], topGO [10]) programming languages. The popularity of such approaches is highlighted by the fact that the initial GOC publication [11] is cited by over 22′000 papers (according to Google Scholar as of October, 2018).

However, the output of current GO analysis web applications (like AmiGO or DAVID) does not fully convey the hierarchical structure of the terms. Tools like GOrilla and NaviGO allow visualization of GO terms’ hierarchy but they in turn lose the relation of GO terms to the genes or proteins being analyzed. Addressing both visualization of term hierarchy and gene-term relations was the main motivation for creating the open source web-application, GOnet (https://github.com/mikpom/gonet). It is achieved by generating a fully interactive graph with gene and term nodes. The graph supports different layouts making it possible to extend analyses based on graph topology.

Occasionally, a researcher might need to go through the functions of each investigated gene products to get more granular information. For such per-entry analysis the researcher might need to retrieve information from various public resources. GOnet complies with this approach and provides convenient links to external databases (UniProt [12], Ensembl [13], DICE-DB [14], Genecards [15]) in the resulting view. In addition, expression data from external sources can be used to colorize gene nodes and provide further insight into the signature investigated. Overall these features make GOnet an important tool to facilitate biological interpretation of -omics data for experimental and computational biologists.

## Implementation

### User’s workflow

**Fig. 1:**
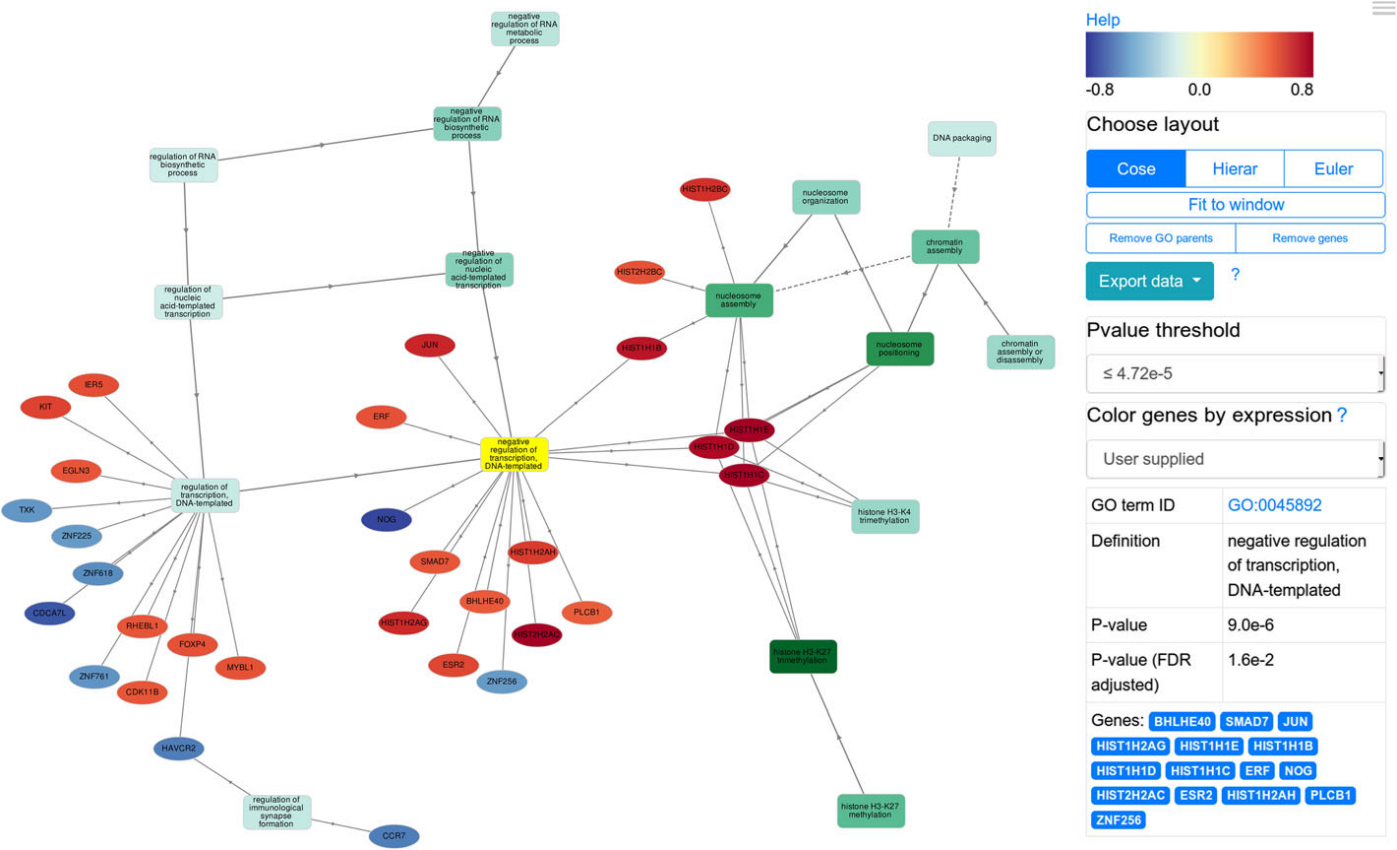
Sample network output generated by GOnet application. Gene differentially expressed in CD4 Bulk Memory T cells in Latent TB patients compared to healthy controls were used as an example [22]

In a basic workflow, the GOnet application receives a list of gene symbols, protein symbols, or protein IDs (UniProt IDs) as an input, and outputs a graph (an example given in Fig. 1). There are various input parameters which will affect the actual structure of the graph visualized and its appearance. The first main user choice is which GO terms the genes are annotated against:

1. GO terms statistically significantly over-represented in the gene list submitted.
2. A predefined subset (also known as ‘GO slim’), or a user-supplied list of terms.

In the first case the analysis will be referred to as an ‘**enrichment’** analysis, in the second as an ‘**annotation’** analysis.

#### Input parameters

1. **Gene list.** A mandatory input parameter containing the genes/proteins of interest. Currently human and mouse data is supported. An example of a human gene list might look like this:

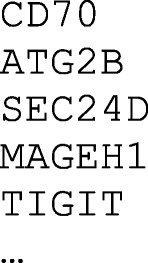

    The gene list can also be accompanied with a contrast value. For example,

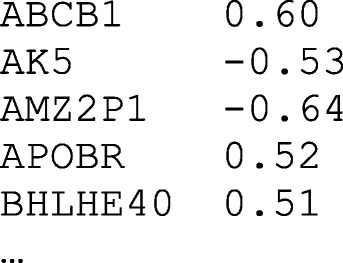

    This contrast value can be any decimal number, such as the log-fold change of gene expression between two conditions. This is merely a visualization enhancement. If the value is supplied it can be used later to differentially color specific genes in the graph (note different colors of gene nodes in Fig. 1), and visually indicate up- or down-regulation of specific genes and gene clusters.
    The application can process common gene symbols (like in the example above), UniProt IDs, and MGI Accession IDs (mouse only). The former type of ID (gene symbols), although is the most human friendly, can unfortunately be ambiguous. For example, AIM1 can mean ‘absent in melanoma’ (also called CRYBG1) or ‘Aurora and Ipl1-like midbody-associated protein’ (also known as AURKB). Due to this ambiguity UniProt IDs or MGI accession IDs (for mouse) are preferred.
2. **GO namespace.** Can be any of ‘biological process’, ‘molecular function’ or ‘cellular component’. Keeping analysis of the three domains separate simplifies the output graph.
3. **Analysis type.** Can take value of ‘enrichment’ or ‘annotation’.
4. **Background** (‘enrichment’ analysis only). A baseline set of genes which the signature is analyzed against. As a background a user can indicate to use a) all annotated genes, b) submit a custom gene list or c) select one of the predefined backgrounds. If the first option is selected the signature will be analyzed versus all genes for which GO annotation information is available. This can serve as a simple default, but the results may not be specific enough. For example, it makes sense to exclude genes not expressed in analyzed cells. A user can upload a list of genes/proteins (same ID types as for the submitted signature are accepted) or select a predefined background. Using the ‘predefined background’ option allows the user to analyze the signature against genes expressed above a value of 1 TPM in one of the cell/tissue types according to expression data available in GOnet (see ‘Technical details of implementation’ section for available expression datasets).
5. **q-value threshold** (‘enrichment’ analysis only). Only GO terms rejected while controlling False Discovery Rate at the value of this parameter will be displayed. To denoise/simplify graph lower parameter values should be considered. Available choices are: 0.05 (also commonly denoted as *), 0.01 (**), 0.001 (***) and 0.0001 (****).
6. **GO subset** (‘annotation’ analysis only). A subset of Gene Ontology to annotate input entries against. The application will reconstruct the relationship of the input genes to GO terms specified by this parameter. For example, ‘GO slim generic’ can be selected. This is a subset of general GO categories maintained by GOC which may be suitable for the majority of studies. Alternatively, users can select the ‘custom’ option and submit a list of GO terms.
7. **Output type.** Results of the default ‘Interactive Graph’ output type is depicted in Fig. 1 and exhibits the main advantage of the GOnet application. If the interactive output is not required then ‘CSV’ option can be selected and the output will be a regular machine-readable text file. In this scenario the application does not reconstruct the graph saving computational time. As an intermediate solution ‘TXT’ output option can be selected. This is a human-readable text file which attempts to retain hierarchical relationship between GO terms in a textual representation.

#### Capabilities of the graphical output

The output graph is interactive (rendered within Cytoscape.js framework [16]) and allows researcher to re-arrange genes and GO term annotations so that they optimally represent the interpretation of the discovered functional classification pattern. There are several features available in the side panel which can assist in graph re-arrangement. Usage experience will be different depending on the number of nodes in a graph (genes nodes as well as the GO term nodes) and their connectivity. If output has a lot of gene nodes, they can be hidden to explore GO terms only. Alternatively, if output contains too many GO term nodes (like in some cases of enrichment analysis) then varying *p*-value thresholds can be applied to narrow down to the most significantly enriched categories.

Depending on the nodes being visualized various layouts can be applied.

1. COSE (Compound Spring Embedder) layout. This layout imitates node repulsion. It is convenient for small graphs containing not many genes (150 or less). This layout is depicted in Fig. 1. Layout implementation is bundled with Cytoscape.js library.
2. Hierarchical layout. This layout displays nodes in their hierarchy. Less specific GO terms are placed at the top of the graph while more specific GO terms are placed at the bottom. Genes (if visualized) are positioned at the lowest level of graph hierarchy. This layout is especially useful for large graphs containing many GO terms. Layout is implemented using cytoscape-dagre JS package.
3. Euler layout. Another force-directed (physics simulation) layout which is similar to COSE layout but runs faster and is more suitable for large graphs. Layout is implemented using cytoscape-euler JS package.

#### Data export

Depending on downstream manipulations the user can choose one of the available data export options:

- Text formats
  - Data as comma-separated file. This is the main machine-readable output format containing the terms, their *p*-values of enrichment (if applicable), and corresponding genes.
  - Data as text file. This format attempts to retain hierarchy of the enriched terms and can be viewed in any text editor.
  - ID mapping. This option allows the user to download a text file with resulting conversion of user input to external database IDs: UniProt, Ensembl, MGI (if applicable).
- Images
  - Image of visible area can be exported in PNG or JPG formats.
- JSON
  - Graph can be downloaded in .cyjs format. CYJS files ca be viewed in the desktop Cytoscape application [17].

#### Contextual menu and node data

The main advantages of GOnet become apparent when a moderate (< 150) number of genes or proteins is submitted to the application. Such concise signatures can be analyzed on a per-entry level. For this purpose, all elements in the graph are clickable and invoke contextual data fields in the side panel showing related information. If the clicked element is a **GO term node** then the information listed includes the term ID (with link to GO database), *p*-value of enrichment (if applicable), and all the entries submitted which are annotated with this term. If a **gene node** is clicked then the side panel provides links to UniProt, Ensembl, DICE-DB, Genecards, and MGI (for mouse genes) databases and all GO annotations of a gene. If an **edge** connecting a gene and GO term is clicked, the corresponding GO references are listed. If an **edge** connecting two GO term is clicked, the relation type is shown (currently ‘is_a’ and ‘part_of’ relation types are supported).

Right clicking on a node invokes a contextual menu which allows the user to select immediate or all successors/predecessors of the node. This highlights all genes/terms downstream of a certain category that the researcher wishes to narrow down to and explore separately.

### Technical details of implementation

**Fig. 2:**
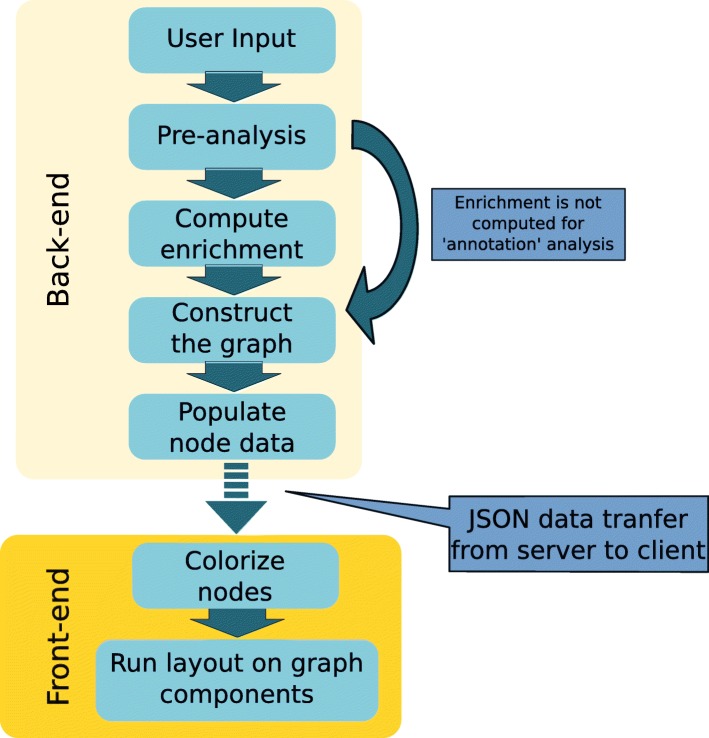
General workflow of GOnet application

The general outline of the steps being implemented by the program is illustrated in Fig. 2. Graph construction is carried out on the server side. The back-end is implemented in Python with Django package as a web framework [18]. The calculated graph with associated data is serialized to JSON and transferred to the client side where the front-end implements layout rendering and node visualization. The Cytoscape JavaScript library [16] is used for visualization.

The workflow is as follows:

1. **Pre-analysis.** Post submission input checks and ID conversion are carried out at this step. Overall strategy of ID conversion is the following: entries submitted by the user are first converted to species-specific primary IDs and then these primary IDs are converted to other IDs. UniProt IDs and MGI Accession IDs are used as primary IDs for human and mouse data respectively. If the user submits UniProt ID for human and MGI IDs for mouse then no conversion to primary IDs is attempted. At every ID mapping step, the application tries to establish 1-to-1 mappings by picking the most relevant and reliable ID possible. For example, in the case of several UniProt IDs, those belonging to SwissProt subset will be preferred because this subset is constructed out of the most reliable records [12]. In the case of duplicated Ensembl IDs, those located on regular chromosomes are prioritized over those located on assembly patches and alternative loci. These restrictions are aimed at providing the user with the most concise and reliable information possible while at the same time trying not to obscure biological interpretation with vast numbers of (sometimes redundant) cross-references. Final ID mappings can be downloaded from the results page. Those entries for which ID conversion has failed will still be visible in the graph but corresponding GO and/or expression information will be missing.
2. **Compute enrichment.** Computation of enrichment *p*-values follows the algorithm in the Python goenrich package [8]. For every GO term considered, the *p*-value in Fisher exact test is computed. For every term, the null hypothesis states that the number of genes in the input list annotated with the GO term is not overrepresented compared to the background. The contingency table considered is:
  **Table:** 

	Entries in background and **in** input list	Entries in background but **not in** input list	Total
**Annotated** with GO term	x	n-x	n
**Not annotated** with GO term	N-x	M-N-(n-x)	M-n
Total	N	M-N	M
    Then the *p*-value is computed as a survival function of hypergeometric distribution with shape parameters (M, n, N) at point x. Next, all *p*-values are subject to FDR control procedure [19]. Those GO categories for which FDR procedure rejects the null hypothesis are carried over to the next steps.

3. **Construct the graph**. At this step the application constructs a NetworkX [12] Directed Graph with submitted entries and GO terms. The graph construction procedure is subject to the following constraints:
  Two GO terms are connected with an edge if they are directly connected in Gene Ontology (by ‘is_a’ or ‘part_of’ relationships). The edge is directed from the more general term to the more specific term.
  Genes are connected to the most specific GO term possible. For example, in Fig. 1, histones HIST1H1C, HIST1H1D, and HIST1H1E are connected to ‘nucleosome positioning’ and not to the more general category of ‘nucleosome organization’. Edges are always directed from GO term to gene.
  Nodes not connected to anything are left as orphan nodes.
  Since two types of GO term relations are used (‘is_a’ and ‘part_of’) it introduces ‘redundancy’ in the graph. Some of the edges can be removed so that if a directed path between any pair of GO term nodes exists in the original graph, then some path between these terms will exist in a reduced graph. Such a reduced graph is constructed using a transitive reduction algorithm on the graph from the previous step. Next, necessary data is added to the graph elements.
4. **Populate node data.** At this step additional information about graph elements is being stored as node or edge attributes. This includes various IDs (UniProt ID, Ensembl ID, MGI ID), expression data, GO references, etc.

After this step the graph is converted to cyjs format (a flavor of JSON specifically adapted for use in Cytoscape applications) and transferred to the client for visualization.

5. **Colorize nodes.** Two different color maps are applied to GO term nodes and gene nodes. The intensity of GO term node colors indicates *p*-values of enrichment. The colors of gene nodes indicate expression values. These values can be supplied as contrast values during the submission process. Alternatively, one can use expression values available from currently supported datasets. For human genes the following expression data are supported:
1) DICE-DB (http://www.dice-database.org/) data. Dataset covers major blood cell types [14].
2) Human Protein Atlas data. Dataset is available at https://www.proteinatlas.org/ [20] and covers major human tissues.
  For mouse genes expression data used is taken from
3) Bgee database [21].

6. **Run layout.** Nodes of the graph are split into connected components; then a user specified layout is applied to every component. All orphan nodes (not connected to any other node) are positioned separately on a grid.

ID resolution, GO analysis, and node data population involves various data sets from external databases which are subject to updates of various frequency. New versions of the corresponding data files are incorporated every two months.

## Results and discussion

The application of genome-wide experimental approaches to biological problems has raised the challenge of how the resulting data can be fully utilized. Computational methods can help to grasp otherwise immense high-throughput data. Several databases and related applications exist for this purpose. Namely, the Gene Ontology database provides an extremely important utility to filter down the complexity of -omics data. Various available GO tools facilitate biological classification of the provided gene lists and help to highlight over-represented functional groups. However, in practice, this is a starting point for further analysis in which a biologist uncovers an underlying biological effect leading to these observations. This transition from data to biological interpretation can be complex and various visualization techniques are especially useful at this step. In the case of Gene Ontology analysis, the hierarchy of the vocabulary can be conveniently visualized as a graph. This graph-based approach was utilized by GOnet application for Gene Ontology analysis. Additionally, the tool provides several features especially useful for users working with genomic/transcriptomic/proteomic data and will help to adapt GO vocabulary to their research needs.

GOnet specifically aims to construct and display interactive graphs that include GO terms and genes while retaining term-gene relationships. Interactivity of a graph gives easy access to node and edge data linking the entries to external databases. It provides the possibility of one-click access to gene/protein data available in UniProt, Ensembl, DICE-DB, Genecards, and GO term data available in AmiGO.

Depending on the size and structure of the graph, the application allows the user to arrange and filter the nodes to adapt the graph further for particular use cases. Specifically, several layouts can be applied depending on what information the user wants to highlight. If GO term hierarchy is the main focus, then a hierarchical layout can be applied which positions terms depending on their ‘is_a’ and ‘part_of’ relationships. Gene nodes can be completely hidden in this case. If one needs to highlight gene-term relationships, then physics simulation layouts imitating node repulsion can be applied. A refined arrangement of the nodes can be exported for illustrative purposes.

Another important advancement of the application is integration of two different yet related tasks: GO enrichment analysis and GO annotation analysis. In the first case, a user is interested in which functional categories are enriched in a specific list of genes or proteins. In the second case, the user’s intent is to have a general look at the categories present in the list regardless of the enrichment score. In both of these tasks, the goal is to browse how a list of genes or proteins is related to a certain subset of GO vocabulary. The difference is in which terms will constitute this subset. Due to the inherent similarity of the two tasks, they can be implemented within a single framework. Additional input parameters can specify GO subsets further, and for the enrichment analysis, the user can limit GO terms by imposing an FDR procedure threshold. For the annotation analysis, the user can choose a certain GO subset to analyze against or even supply a custom subset of the Ontology. Currently, the application supports a generic GO slim maintained by the GOC but we believe that creation of such subsets is an important direction for further adapting GO tools to specific research areas.

GOnet also provides transparent ID conversion. The user can check on a per gene level how the input entries were converted to external database IDs. If the conversion is not satisfactory, the user can make changes to the input accordingly by incorporating specific primary IDs (UniProt ID for human and MGI IDs for mouse) where necessary. Primary IDs are unambiguous and generally lead to more consistent results.

Lastly, the application supports various export options valuable for downstream analysis. These options include machine readable delimiter-separated files and JSON-serialized files suitable for analysis in desktop versions of the Cytoscape application.

## Conclusions

Researchers working with -omics data often face the problem of biological interpretation of a list of genes or proteins they obtained from upstream analysis steps. Utilizing a Gene Ontology annotation/enrichment approach is very useful at this stage, but several advancements can be made to improve interpretation of such data. Specifically, one could benefit from interactive analysis of relationships between the entries and their GO annotations. Here we present a GOnet tool which implements such interactive analysis in the form of a web application. On top of that, GOnet has several additional features facilitating per-entry review of the data by providing links to external databases containing biological information about the submitted entries. We believe the application can help to summarize and explore -omic data in a convenient and informative way.

## Abbreviations

FDR: False Discovery Rate
GO: Gene Ontology
GOC: Gene Ontology Consortium
GSEA: Gene Set Enrichment Analysis
TPM: Transcripts per Million
